# Spontaneous Documentation of Bidirectional Block During Pulmonary Vein Isolation - Keep an Eye on the Electrograms!

**DOI:** 10.1016/s0972-6292(16)30694-5

**Published:** 2013-11-15

**Authors:** Stylianos Tzeis, Sokratis Pastromas, George Andrikopoulos

**Affiliations:** Cardiology Department, Henry Dunant Hospital, Athens Greece

**Keywords:** bidirectional block, pulmonary vein, ablation, atrial fibrillation

## Abstract

In the present case, we describe the abrupt transformation of intra-pulmonary vein activity from rapid firing to dissociated ectopic activity during sinus rhythm, as an easily identifiable, though rare to encounter, sign which documents the achievement of bidirectional block.

A 47-year old patient underwent a catheter ablation procedure due to recurrent, symptomatic, drug-resistant episodes of atrial fibrillation. The ablation strategy included pulmonary vein (PV) isolation by deployment of circumferential lesions surrounding the PVs with an irrigated ablation catheter (Navistar Thermocool SF, Biosense Webster, inc., Diamond Bar, CA) and the use of a three-dimensional electroanatomical mapping system (CARTO-3). A circular mapping catheter (Lasso, Biosense Webster, Inc., Diamond Bar, CA) was placed within each of the PVs to guide ablation based on the recorded PV potentials.

During the procedure, the right superior PV was highly arrhythmogenic with frequent firing. After a 20-minute waiting period following initial isolation of all PVs, we controlled each vein for potential resumption of electrical connection with the left atrium. The figure (Figure 1) presents recording from the circular catheter located in the right superior PV during sinus rhythm. In the left part of the recording, the circular catheter records fibrillatory activity within the PV. However, electrical impulses from the PV are not conducted to the left atrium (LA), as evidenced by the uninterrupted sinus rhythm on the surface ECG and the regular activity in the decapolar catheter placed in the coronary sinus. The interruption of impulse propagation from the PV to the LA demonstrates the achievement of exit block.

In the right part of the recording the rapid intra-PV firing ceases abruptly and a dissociated slow ectopic intra-PV activity is recorded. The measured intervals between atrial activation and PV spikes, as shown in the figure, are not constant, supporting the presence of dissociated PV ectopy and entrance block. Adenosine was not administered due to coexistent contraindication.

It should be noted that apart from the dissociated PV rhythm, the circular catheter also records residual potentials. These electrograms are far-field with an origin from the superior vena cava based on the following: (a) they are recorded by the catheter bipoles situated at the superior and anterior part of the vein which is adjacent to the superior vena cava and (b) the timing of the recorded potentials is less than 30 msec from the onset of the P wave, which is a highly sensitive and specific marker to recognize far-field potentials of SVC origin recorded in the right superior PV.[2]

## Discussion

In the present case, we describe the abrupt transformation of intra-PV activity from rapid firing to dissociated ectopic activity during sinus rhythm, as an easily identifiable, though rare to encounter, sign which documents the achievement of bidirectional block.

Based on the recently issued expert consensus statement on catheter and surgical ablation of atrial fibrillation, electrical isolation of PVs is the primary goal for most ablation procedures.[1] The concept of this ablation strategy is to interrupt the electrical connections of arrhythmogenic triggers located at the ostium or within PVs with the atrial myocardium. The accomplished electrical disconnection prevents focal firing of PV sources from triggering AF paroxysms. A critical step in the AF ablation procedure is the verification of PV isolation, usually with the aid of a circular mapping catheter located within the PV. Reliable and timely identification of PV isolation has profound clinical implications in terms of increased treatment efficacy but also enhanced procedural safety. Failure to recognize PV isolation results in redundant ablation and therefore higher likelihood of collateral damage and periprocedural complications.

The most stringent endpoint of PV isolation is the demonstration of bidirectional conduction block, meaning the interruption of conduction from the LA to PV (entrance block) as well as in the opposite direction from PV to LA (exit block). From a pathophysiological perspective, it is more important to document conduction block from the PV to LA (exit block) since this is the direction of impulse conduction when a rapidly firing PV focus triggers AF paroxysms. However, as stated in the expert consensus statement, only 10% of the task force members rely on the documentation of exit block as an endpoint of the ablation procedure, while 75% use only entrance block as a primary endpoint of PV isolation.[1] 

Entrance block is verified by elimination of PV potentials or by recording dissociated PV rhythm (isolated ectopy or slow regular rhythm). Dissociated PV potentials represent intra-PV electric discharges which are independent from left atrial electric activity (parasystole-like).

Exit block can be demonstrated with or without the use of pacing maneuvers. The concept of electrophysiologic maneuvers is to pace within the PV at a rate faster than the sinus rate and to demonstrate the inability of the captured intra-PV impulses to propagate to the left atrium. The limitation of this maneuver is the difficulty to achieve reliable local PV capture. Therefore, pacing at high output is needed frequently, which though may result in inadvertent far-field capture of neighboring anatomic structures and thus to an erroneous diagnosis of persistent PV to LA connection.[3] Exit block can also be spontaneously demonstrated in the presence of an ectopic PV rhythm. If the PV rhythm is faster than the sinus rhythm (fibrillatory activity or PV tachycardia) then the documentation of exit block is based on the absence of temporal relationship between the PV and LA activity. In the case of a slow PV rhythm or isolated PV ectopy, the documentation of exit block is more challenging, since advancement of LA activation by the dissociated PV potentials needs to be ruled out.[4]

The verification of conduction block at a certain direction should not be considered synonymous to bidirectional block. The term "unidirectional block" has been used to describe the persistence of PV-LA conduction (absence of exit block) despite the proof of LA-to-PV conduction (entrance) block or vice versa. However, the reported occurrence rates of unidirectional block vary in the literature from 0.6% to 42%, reflecting differences in implemented ablation strategy (segmental vs. circumferential), methodological issues and use of differential pacing maneuvers.[3-5]

In conclusion, the achievement of bidirectional block is the sine-qua-non during PV isolation. The presented sign verifies spontaneously bidirectional block and the electrical disconnection of pulmonary veins.

## Figures and Tables

**Figure 1:**
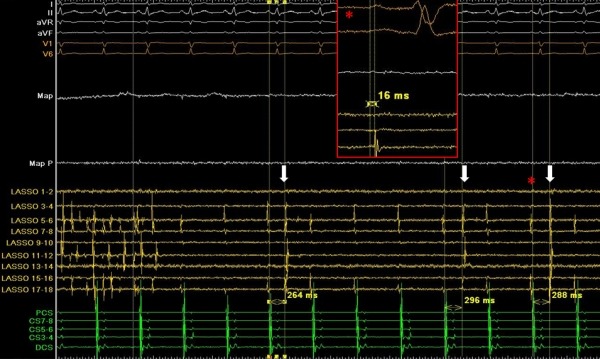
Recording of right superior pulmonary vein (PV) after isolation. Spontaneous transformation of intra-PV activity from rapid firing to dissociated ectopy validates the achievement of bidirectional block. In the left part of the recording, the circular catheter records fibrillatory activity within the PV during uninterrupted sinus rhythm, documenting exit block. In the right part of the recording, an intra-PV ectopy is recorded (white arrows). The measured intervals between atrial activation and PV spikes are not constant, supporting the presence of entrance block. In the red caption (enlarged view of the part of the recording denoted with a red asterisk) please note the temporal relationship of the residual potentials recorded in the circular catheter with the onset of the sinus P-wave (< 30 msec), which identifies their extra-PV origin (far-field electrogram from superior vena cava). From top to bottom surface ECG leads (I, II, aVR, aVF, V1, V6), distal bipole of the ablation catheter (Map), circular mapping catheter bipoles (LASSO1-2 to LASSO 17-18) and bipoles of a decapolar catheter placed in the coronary sinus. PCS: proximal coronary sinus bipole, DCS: distal coronary sinus bipole.
